# Overweight and obesity in non-pregnant women of childbearing age in South Africa: subgroup regression analyses of survey data from 1998 to 2017

**DOI:** 10.1186/s12889-022-12601-6

**Published:** 2022-02-25

**Authors:** Mweete Debra Nglazi, John Ele-Ojo Ataguba

**Affiliations:** 1 Health Economics Unit, School of Public Health & Family Medicine, Faculty of Health Sciences, University of Cape Town, Cape Town, 7925 South Africa; 2 Department of Community Health Sciences, Max Rady College of Medicine, Rady Faculty of Health Sciences, University of Manitoba, Winnipeg, Canada

**Keywords:** Overweight, Obesity, Non-communicable diseases, Non-pregnant women, South Africa

## Abstract

**Background:**

Overweight and obesity in adults are increasing globally and in South Africa (SA), contributing substantially to deaths and disability from non-communicable diseases. Compared to men, women suffer a disproportionate burden of obesity, which adversely affects their health and that of their offspring. This study assessed the changing patterns in prevalence and determinants of overweight and obesity among non-pregnant women in SA aged 15 to 49 years (women of childbearing age (WCBA)) between 1998 and 2017.

**Methods:**

This paper conducts secondary data analysis of seven consecutive nationally representative household surveys—the 1998 and 2016 SA Demographic and Health Surveys, 2008, 2010–2011, 2012, 2014–2015 and 2017 waves of the National Income Dynamics Survey, containing anthropometric and sociodemographic data. The changing patterns of the overweight and obesity prevalence were assessed across key variables. The inferential assessment was based on a standard t-test for the prevalence. Adjusted odds ratios from logistic regression analysis were used to examine the factors associated with overweight and obesity at each time point.

**Results:**

Overweight and obesity prevalence among WCBA in SA increased from 51.3 to 60.0% and 24.7 to 35.2%, respectively, between 1998 and 2017. The urban-rural disparities in overweight and obesity decreased steadily between 1998 and 2017. The prevalence of overweight and obesity among WCBA varied by age, population group, location, current smoking status and socioeconomic status of women. For most women, the prevalence of overweight and/or obesity in 2017 was significantly higher than in 1998. Significant factors associated with being overweight and obese included increased age, self-identifying with the Black African population group, higher educational attainment, urban area residence, and wealthier socioeconomic quintiles. Smoking was inversely related to being overweight and obese.

**Conclusions:**

The increasing trend in overweight and obesity in WCBA in SA demands urgent public health attention. Increased public awareness is needed about obesity and its health consequences for this vulnerable population. Efforts are needed across different sectors to prevent excessive weight gain in WCBA, focusing on older women, self-identified Black African population group, women with higher educational attainment, women residing in urban areas, and wealthy women.

## Background

Obesity is a global public health problem. Overweight and obesity among adults increased globally, with obesity prevalence almost tripling since 1975 [1]. In 2016, the World Health Organization (WHO) estimated that over 2 billion adults worldwide were overweight or obese [1], and over 70% of overweight or obese adults resided in low- and middle-income countries (LMICs) [2]. The condition of obesity is largely driven by the obesogenic environment where there is easy accessibility, affordability and availability of high energy-dense foods, preference for the consumption of these foods, in addition to reduced opportunities for physical activity at work, community or leisure [3]. Obesity features among key risk factors for adverse outcomes from the coronavirus disease 2019 (COVID-19) [4, 5].

In South Africa, the prevalence of adult overweight and obesity [6] has increased, and this has been linked to economic growth and nutritional transition [7–9]. Obesity also contributes substantially to deaths and disabilities from non-communicable diseases, including cardiovascular diseases, diabetes and some cancers [10]. The country faces a dual burden of overweight and obesity among adult women aged at least 15 years [11]. Overweight and obesity were respectively implicated in 18 and 57% pulmonary embolism deaths among mothers. The obesity burden in South Africa is disproportionately higher among women than men. According to a government report published in 2019, about 41% of women and 11% of men aged 15 years and above were obese [12]. Because women of childbearing age between 15 to 49 years old (WCBA) accumulate weight faster than other women [13–16], the adverse consequences of obesity among this group could be pronounced. Obesity during a woman’s childbearing years is associated with an increased risk of infertility, miscarriage, stillbirths and births with congenital disabilities, shoulder dystocia and other adverse obstetric outcomes [17–22]. In recognition of the magnitude of obesity, especially among women in South Africa, the government set targets in August 2013 to reduce obesity [23]. For example, it targets reducing the prevalence of overweight and obesity by 10% by 2020 from 1998 (particularly, the overweight and obesity prevalence for adult women aged 15 years and above in 1998 were 56 and 30%, respectively) [23, 24], and this requires not only evidence but a clear plan of action.

Studies in the United States [25], Morocco [26] and sub-Saharan Africa [27, 28] show obesity prevalence among WCBA ranging between 10 and 39%, with rates over 30% in urban Egypt [27] and South Africa [28]. Recent studies have shown an increasing trend in overweight and obesity among WCBA in sub-Saharan Africa [27, 29–33] and Bangladesh [34]. Research also shows that the prevalence varies by subgroups (age groups, educational attainment, socioeconomic status, parity and race/ethnicity) [25, 31–33, 35], although patterns might differ between high-income countries and LMICs. For example, in high-income countries, obesity prevalence is higher among women with low education and the poor [25], while the reverse pattern is seen in LMICs [31–33, 36]. In sub-Saharan Africa and elsewhere, factors such as increased age [25, 37], increased parity [38], being rich [29, 36, 37], higher education [25, 36–38], urban residence [37], race/ethnic differences [25, 38] and increased television watching or a sedentary lifestyle [38, 39] are associated with a higher probability of overweight and obesity in WCBA. In South Africa, apart from earlier studies (including a government report) showing the prevalence of overweight and obesity at one single point in time [28, 40, 41], there is a dearth of studies looking at trend data on overweight and obesity prevalence among WCBA, including their socioeconomic correlates or determinants.

This study is critical, mainly as the country faces a burgeoning threat of non-communicable diseases, especially among women [42, 43], and there is a desire to address these challenges. South Africa recorded about 119 maternal deaths per 100,000 live births in 2017 [44], which is far higher than the target set for the sustainable development goal (i.e. reducing maternal mortality ratio to fewer than 70 maternal death per 100,000 live births) [45]. The significant contributions of overweight and obesity to maternal morbidity and mortality in South Africa have been documented. Obesity led to significant pregnancy complications, including hypertensive, pre-eclamptic and surgical complications [46], including lower quality of life and distress [47]. It is critical to understand the evolution in prevalence and related determinants of overweight and obesity over the last decades in the South African setting. Answering this research question will inform policy targeted action/interventions to reach the national obesity targets [23]. This study, therefore, assessed, for the first time in South Africa, the change in the prevalence of overweight and obesity among non-pregnant WCBA between 1998 and 2017. It also identified the determinants of overweight and obesity in this population.

## Methods

### Data sources

This paper was based on a secondary analysis of de-identified data from several national household surveys that received ethics approval. These anonymised datasets are publicly available and hosted on the DataFirst portal at the University of Cape Town (www.datafirst.uct.ac.za). The nationally representative datasets used in this paper, detailed below, include the 1998 and 2016 South Africa Demographic and Health Surveys (SADHS), and the 2008–2017 National Income Dynamics Study (NIDS). These data are comparable because they are nationally representative and used a similar sampling strategy.

### South Africa demographic and health surveys

The 1998 and 2016 SADHS are nationally representative cross-sectional surveys. The fieldwork for the 1998 SADHS was between January and September 1998, with a total sample size of 11,735 women (i.e. a 95% response rate among women) [24]. The 2016 SADHS was undertaken between July and September 2016 yielding a total sample size of 8514 women (i.e. a 86% response rate) [12]. The sampling procedures for the SADHS are detailed elsewhere [12, 24]. Briefly, the 1998 and the 2016 SADHS used a two-stage sampling strategy with the 1996 and 2011 Census Enumeration Areas (EAs) as sampling frames, respectively. The EAs were stratified into the nine provinces and by urban, farm and traditional areas. The first stage consisted of selecting EAs with probability proportional to the size. The second stage consisted of systematically sampling residential dwelling units. The SADHS collect information from a household questionnaire, biomarker questionnaire, woman’s questionnaire and a man’s questionnaire. The DHS data are stored in several dataset files: household recode, individual recode, birth’s recode, kid’s recode, men’s recode and couples recode. This paper used data from the women and household’s files.

### National Income Dynamics Study

The NIDS is a nationally representative longitudinal panel survey repeated every two years since 2008 by the Southern African Labour and Development Research Unit, funded by the South Africa Presidency. Fieldwork for the first NIDS wave (2008) was carried out between February and December 2008. The second wave (2010–2011) between May 2010 and September 2011. The third wave (2012) was between May and December 2012. The fourth wave (2014–2015) was between September 2014 and August 2015, and the latest wave (2017) was between February and December 2017 [48]. The overall survey response rates for the 2008, 2010–2011, 2012, 2014–2015 and 2017 waves were 51.2, 45.3, 49.9, 53.9 and 53.3%, respectively. Details of the sampling procedure, including calculating the different sampling weights, are described elsewhere [48–51]. Briefly, the NIDS used a stratified two-stage cluster sampling strategy to sample households at baseline. A total of 400 primary sampling units (PSUs) were selected in the first stage from Statistics South Africa’s 3000 PSUs in the 2003 Master sample. In 2008, a total of 7305 households were interviewed in the 400 PSUs. All household members became a Continuing Sample Member (CSM) to be interviewed every two years. Children born to CSM women after Wave 1 are ‘born into’ the sample. Everyone currently living with a CSM (i.e. individuals referred to as Temporary Sampling Members [TSMs]) are also interviewed. As CSMs move out and start their households, the number of interviewees also grows. Trained fieldworkers collected the data through standardised questionnaires across the waves; household questionnaire, adult questionnaire for adults aged 15 years and older, proxy questionnaire for non-available adults, and child questionnaires for children aged between 0 and 14 years. This paper used data from the 2008–2017 NIDS waves based on the adults (containing WCBA) and household questionnaires.

### Participants

The analysis included adult non-pregnant women aged between 15 and 49 years (i.e., WCBA). This definition includes lactating women.

### Definitions of key variables

Table 1 contains a description of the key variables used in this paper.

**Table 1:** A description of key variables used in the analysis

Variable	Definition
Overweight	A body mass index (BMI) ≥ 25 kg/m^2^ [52]
Obesity	A BMI ≥ 30 kg/m^2^ [52]
Age category 1	A woman aged between 15 years and 24 years
Age category 2	A woman aged between 25 years and 34 years
Age category 3	A woman aged between 35 years and 49 years
Black African^1^	Women self-identified as black African race
Non-black African	Women self-identified as white, coloured and Asian race
No schooling/primary education^3^	A dummy variable for a woman with no education or only primary education
Secondary education	A dummy variable for a woman with secondary education
Tertiary education^4^	A dummy variable for a woman with tertiary education
Rural	A woman residing in a rural location
Urban	A woman residing in an urban location
Quintiles of socioeconomic status^5^	Quintile 1 = 1 if a woman is in the poorest socioeconomic group; 0 otherwise Quintile 2 = 1 if a woman is in the second poorest socioeconomic group; 0 otherwise Quintile 3 = 1 if a woman is in the middle socioeconomic group; 0 otherwise Quintile 4 = 1 if a woman is in the second richest socioeconomic group; 0 otherwise Quintile 5 = 1 if a woman is in the richest socioeconomic group; 0 otherwise
Current smoking status	No = 0 if woman does not currently smoke tobacco including cigarettes Yes = 1 if woman currently smokes tobacco including cigarettes

*Notes*^1^:The self-identified black race group dominates in South Africa with previous studies reporting the existence of racial disparities for obesity and overweight [53]; ^3^No schooling and primary education are combined due to small numbers^4^; Tertiary education refers to education attained post-secondary school. This includes certificates, diplomas, bachelors, master’s and doctoral degrees^5^;Quintiles of socioeconomic status are based on household expenditure per capita for the NIDS datasets and household wealth index for the SADHS datasets

### Dependent variable

According to the WHO, and as used in the present study, overweight and obesity in adulthood were classified as BMI of ≥25 kg/m^2^ and ≥ 30 kg/m^2^, respectively [52] (Table 1).

### Explanatory variables

Guided by the social determinants of health concept [54], several predictors of overweight and obesity were selected (see Table 1). Previous literature, the availability of the variables in the datasets, and the comparability of variables across datasets also guided the selection of predictors. Explanatory variables, as shown in Table 1, include age and population groups, education, area of residence, smoking status and socioeconomic quintiles. Population group was categorised as Black-African or non-black African because self-identified black race group dominates in South Africa with previous studies reporting the existence of racial disparities for obesity and overweight [53].

### Statistical analysis

There was a nationally representative sample of 5403 WCBA in the 1998 SADHS, 7298 women in the 2008 NIDS, 7713 women in the 2010–2011 NIDS, 8683 in the 2012 NIDS, 9703 in the 2014–2015 NIDS and 8210 in the 2016 SADHS and 10,267 in the 2017 NIDS. We computed the descriptive statistics for continuous and categorical data. The continuous variable (i.e., age) was expressed as mean and standard deviation (SD). The categorical variables were expressed as percentages. Descriptive statistics were estimated by subgroup (i.e., population group, education, area of residence, current smoking status and socioeconomic quintile). The prevalence of overweight and obesity for WCBA across the key variables in Table 1 (except for smoking status) and the corresponding 95% confidence intervals (CIs) were estimated in each survey year. The patterns of the prevalence of overweight and obesity were assessed between periods. Also, an inferential assessment using simple t-statistics was used to assess if the prevalence of overweight or obesity across the key variables (except for smoking status) was statistically different between periods [55].

The effect of each explanatory variable on BMI status was examined using multiple logistic regression models. In the multiple logistic regression analysis, the reference group for the dependent variable (not being overweight or obese) was compared to other categories (obese vs not obese; and overweight vs not overweight). The significance of each explanatory variable in predicting overweight or obesity was determined using the likelihood ratio test, which gives *p*-values for each predictor in the model. The outputs of the models were presented using adjusted odds ratios (AOR) and 95% CIs. The multiple logistic regression models were adjusted for age, population group, education, area of residence, current smoking status, and socioeconomic quintiles. The level of significance was set at *p* < 0.05.

All analyses were adjusted for the sample design (cluster and sample weight) and performed in Stata version 15 [56].

## Results

### Descriptive summary

The mean age of women was approximately 30 years between 1998 (mean 29.8, SD 9.71) and 2017 (mean 30.3, SD 9.48), with Black Africans forming the predominant population group (Table 2). Over half of the women had attained secondary education, over 60% women resided in urban areas, and about 6.5–11.5% were currently smoking.

**Table 2:** Descriptive statistics of the sample of women of childbearing age between 15 and 49 years by time period, South Africa

Variables	Survey year % 95% CI						
	1998	2008	2010/2011	2012	2014/2015	2016	2017
**Sample**	5403	7298	7713	8683	9703	8210	10,267
**Age, mean (SD)**	29.8 (9.71)	29.7 (9.87)	29.6 (9.88)	29.8 (9.75)	30.0 (9.79)	30.4 (9.80)	30.3 (9.48)
**Population group**							
Black African	78.4	79.5	80.1	80.9	81.7	86.7	82.1
	75.9–80.7	74.3–83.8	74.8–84.6	75.5–85.4	76.9–85.6	84.4–88.7	77.8–85.7
non-Black African	21.6	20.5	19.9	19.1	18.3	13.3	17.9
	19.3–24.1	16.2–25.7	15.4–25.2	14.6–24.5	14.4–23.1	11.3–15.4	14.3–22.2
**Education**							
No school/primary	41.9	18.7	15.6	13.6	10.7	11.0	7.8
	39.9–43.9	16.9–20.7	14.0–17.4	12.0–15.3	9.6–12.0	9.9–12.2	6.9–8.8
Secondary	51.1	67.9	69.9	69.9	71.6	77.2	71.4
	49.2–53.0	65.9–69.8	67.9–71.8	67.9–71.9	7.0–73.4	7.6–7.9	6.9–73.3
Tertiary	7.0	13.4	14.5	16.5	17.6	11.8	20.8
	6.1–8.1	11.4–15.7	12.7–16.5	14.5–18.6	15.9–19.6	10.4–13.4	18.8–22.9
**Area of residence**							
Rural	36.4	37.2	39.8	39.9	39.0	32.8	34.8
	34.9–38.1	32.4–42.3	34.3–45.6	34.6–45.4	34.1–44.1	30.6–35.2	30.4–39.5
Urban	63.6	62.8	60.2	60.1	61.0	67.2	65.2
	61.9–65.1	57.7–67.6	54.4–65.7	54.6–65.4	55.9–65.9	64.8–69.4	60.5–69.6
**Current smoking status**	11.5	8.9	7.8	6.5	7.7	6.9	6.7
**Yes**	10.3–12.9	7.2–10.9	5.9–10.3	4.9–8.6	5.8–10.0	5.8–8.2	5.4–8.1
	88.5	91.1	92.2	93.5	92.3	93.1	93.3
**No**	87.1–89.7	89.1–92,8	89.7–94.1	91.4–95.1	90.0–94.2	91.8–94.2	91.9–94.6
**Socioeconomic status quintile**							
1 (poorest)	14.9	20.1	20.0	19.9	20.0	19.3	20.1
	13.1–16.8	17.7–22.7	17.6–22.7	17.4–22.8	17.6–22.7	16.7–22.2	17.7–22.8
2 (poor)	18.4	19.9	20.0	20.0	19.9	19.9	20.0
	16.6–20.5	17.9–22.1	17.9–22.2	18.0–22.1	18.0–22.0`	18.0–21.9	18.1–22.0
3 (middle)	20.5	20.0	20.1	20.0	20.1	21.1	20.0
	18.6–22.6	17.8–22.4	18,2–22.1	17.9–22.3	18,3–22.0	19.2–23.2	18.3–21.9
4 (rich)	23.7	20.0	20.0	20.0	19.9	20.8	20.2
	21.4–26.1	17.8–22.4	17.7–22.4	18.0–22.2	18.0–22.0	18.7–23.2	18.4–22.1
5 (richest)	22.5	20.0	20.0	20.0	20.0	18.8	19.7
	20.3–24.9	16.3–24.2	16.4–24.2	16.5–24.1	17.3–23.1	16.2–21.8	16.9–22.7

*SD* standard deviation, % percent, 95% CI 95% confidence interval

### Changes in the prevalence of overweight and obesity over time

#### Overall prevalence

**Fig. 1:**
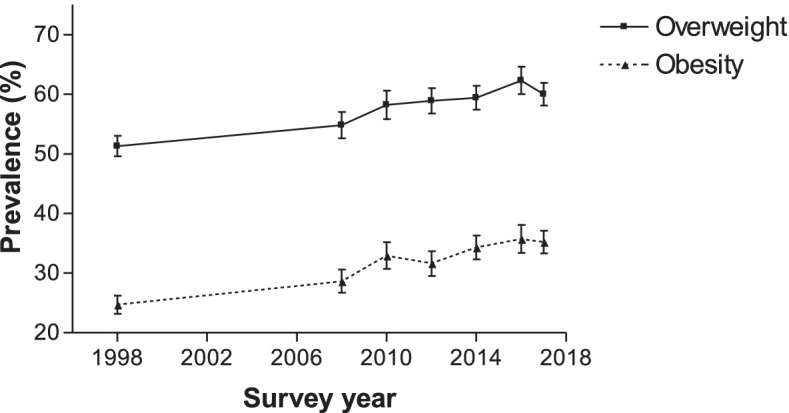
Overweight and obesity prevalence for women aged 15–49 years from 1998 to 2017, South Africa. Error bars represent 95% confidence intervals

The overweight prevalence for WCBA increased from 51.3% (95% CI 49.6–53.0) in 1998 to 62.3% (95% CI 60.0–64.6) in 2016 (*p* < 0.0001), but fell in 2017 (60.0%;95% CI 58.1–61.9) to a lower level than it was in 2016 (*p* = 0.0180). However, the difference of prevalence estimates between 1998 and 2017 were statistically significant (*p* < 0.0001) (Fig. 1). Similarly, obesity prevalence increased from 24.7% (95% CI 23.3–26.2%) in 1998 to 35.7% (95% CI 33.4–38.0%) in 2016 (p < 0.0001), but fell slightly in 2017 (35.2%;95% CI 33.3–37.1%) to a lower level that it was in 2017 (*p* = 0.6411). Here also, the difference of prevalence estimates between 1998 and 2017 were statistically significant (p < 0.0001).

### Prevalence stratified by age group

**Fig. 2:**
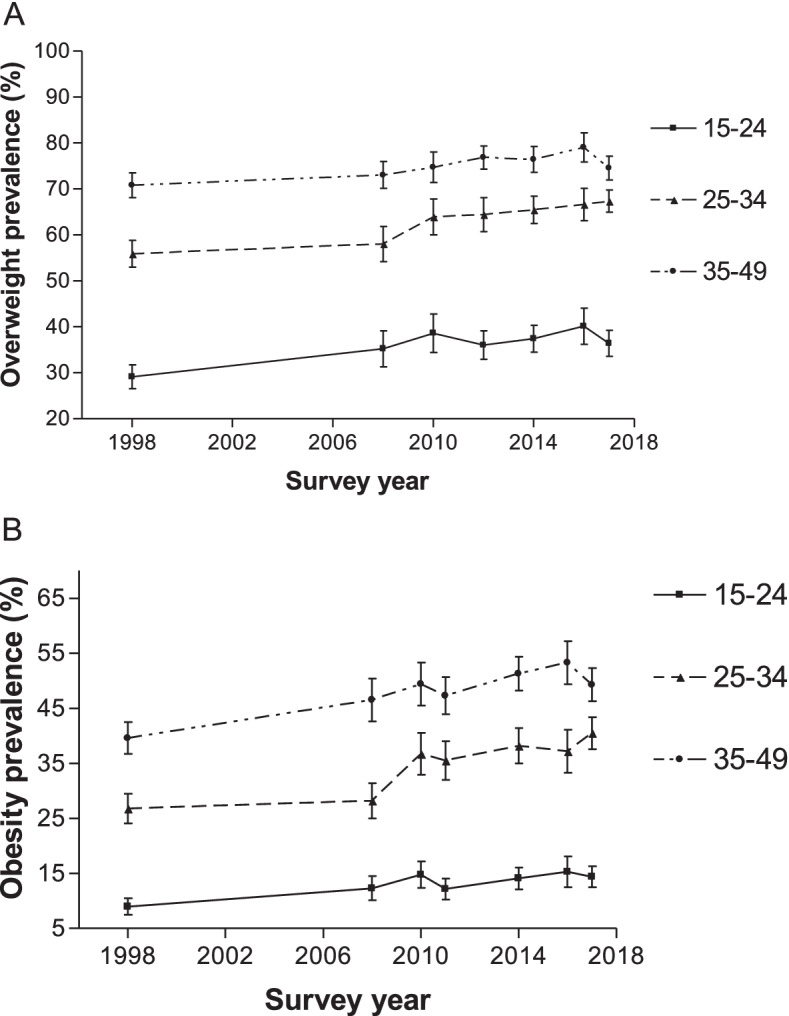
Overweight and obesity prevalence according to age group for women aged 15–49 years from 1998 to 2017. Error bars represent 95% confidence intervals

After stratifying by age group, the overweight prevalence remained higher among older than younger women between 1998 and 2017 (Fig. 2). In 1998, the overweight prevalence was higher among women aged 35–49 years (70.8%) compared to those aged 25–34 years (55.9%) or aged 15–24 years (29.1%). In 2017, the pattern remained similar with the estimate higher among women aged 35–49 years (74.5%) compared to those aged 25–34 years (67.3%) or aged 15–24 years (36.4%). Increasing prevalence of overweight was seen in women aged 15–24 years (29.1% in 1998 to 36.4% in 2017; *p* < 0.0001), 25–34 years (55.9% in 1998 to 67.3% in 2017; p < 0.0001) and 35–49 years (70.8% in1998 to 74.5% in 2017; p < 0.0001), although there were no statistically significant differences for some of the periods.

Similarly, obesity prevalence remained higher among older than younger women between 1998 and 2017 (Fig. 2). In 1998, the obesity prevalence was higher among women aged 35–49 years (39.6%) compared to those aged 25–34 years (26.8%) or 15–24 years (9.0%). In 2017, the estimate was also higher among women aged 35–49 years (49.3%) compared to those aged 25–34 years (40.5%) or 15–24 years (14.4%). Obesity prevalence also increased for women aged 15–24 years (9.0% in 1998 to 14.4% in 2017; *p* < 0.0001), 25–34 years (26.8% in 1998 to 40.5% in 2017; p < 0.0001) and 35–49 years (39.6% in 1998 to 49.3% in 2017; p < 0.0001). However, there were no statistically significant differences in prevalence estimates for some periods.

### Prevalence stratified by population group

**Fig. 3:**
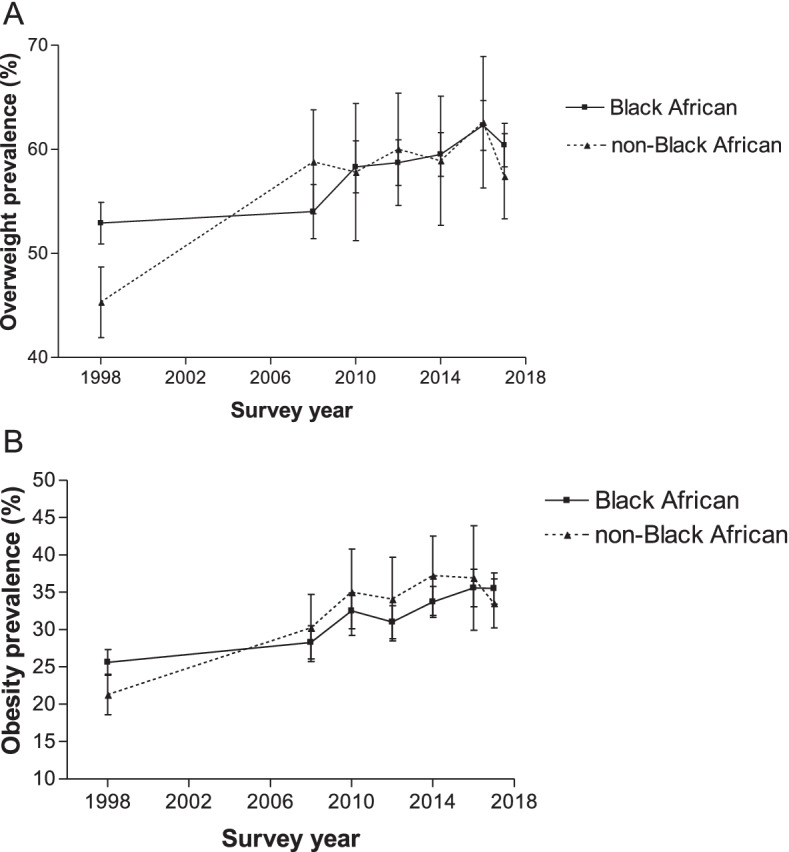
Overweight and obesity prevalence according to population group for women aged 15–49 years from 1998 to 2017. Error bars represent 95% confidence intervals

The results after stratifying overweight and obesity prevalence by population group were different in pattern. In 1998, overweight and obesity occurred more in the self-identified Black African population group than the self-identified non-Black African population group. The gap in overweight and obesity prevalence between the self-identified Black African population and the non-Black African population reduced significantly since 2008 (Fig. 3). In some cases, overweight and obesity prevalence was higher among the non-Black African than Black African population groups.

Generally, except for a few years, the overweight and obesity prevalence among the self-identified Black African and the non-Black African population groups increased between 1998 and 2017 (*p* < 0.0001), though there were no statistically significant differences for adjacent periods. For example, between 1998 and 2017, the obesity prevalence among the self-identified non-Black African population rose from 21.3 to 33.5% (*p* < 0.0001) and from 25.6 to 35.5% (p < 0.0001) among the self-identified Black African population group. Even though the difference in prevalence estimates of obesity and overweight among the self-identified Black African and the non-Black African population groups were not statistically significant for the adjacent years, they were statistically significant when the prevalence in 1998 was compared to that in 2017 (p < 0.0001) (Fig. 3).

### Prevalence stratified by education

**Fig. 4:**
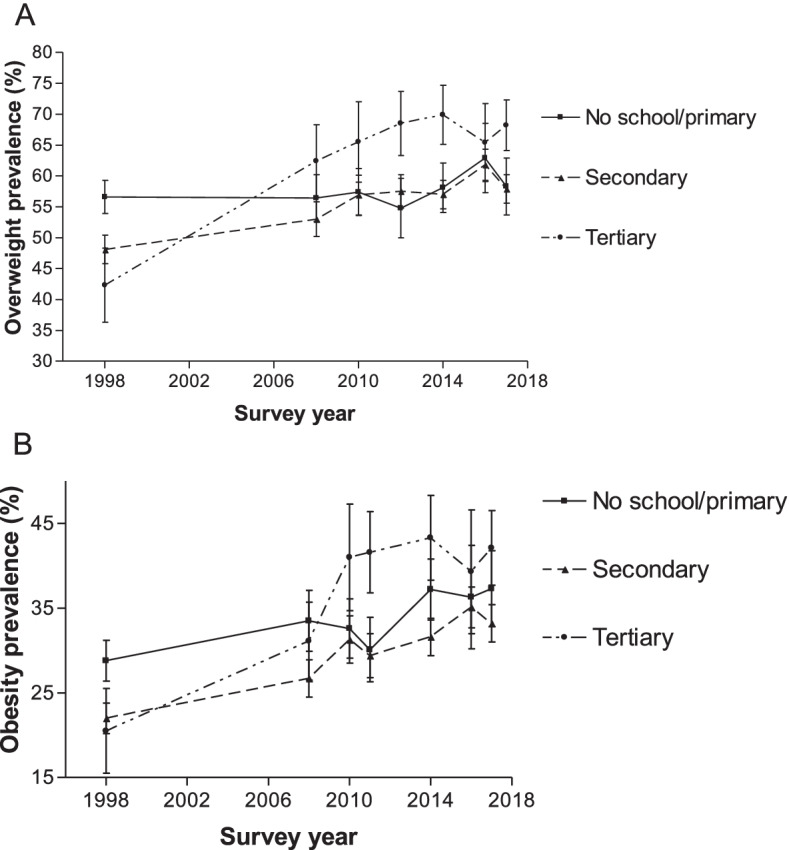
Overweight and obesity prevalence according to education level for women aged 15–49 years from 1998 to 2017. Error bars represent 95% confidence intervals

In 1998, women with no schooling/primary education and women with secondary education had a higher overweight prevalence than those with tertiary education (Fig. 4). By contrast, since 2008, women with tertiary education had a higher overweight prevalence than those with no schooling/primary/secondary education. The overweight prevalence among those with no schooling/primary education has not changed between 1998 and 2017 (56.6% versus 58.3%; *p* = 0.0703) (Fig. 4). In 1998, the overweight prevalence among those with no schooling/ primary education was 56.6% (95% CI of 53.9 to 59.3%). In 2017, the prevalence increased to 58.3% (95% CI of 53.7 to 62.8%). Similarly, the overweight prevalence among women who had a secondary education rose from 48.1 to 57.9% (*p* < 0.0001), between 1998 and 2017. Also, overweight prevalence among women who had tertiary education increased from 42.3% in 1998 to 68.2% in 2017 (*p* < 0.0001)).

Like the overweight results, women with no schooling/primary education and those with secondary education had a higher obesity prevalence than those with tertiary education in 1998 (Fig. 4). However, the pattern changed since 2010 as women with tertiary education had a higher obesity prevalence than those with secondary, primary or no formal education. The trend for obesity was marginally different from that of overweight, where prevalence estimates among women with no schooling/ primary education appeared to have risen between 28.8% in 1998 and 37.3% in 2017 (*p* < 0.0001).

### Prevalence stratified by area of residence

**Fig. 5:**
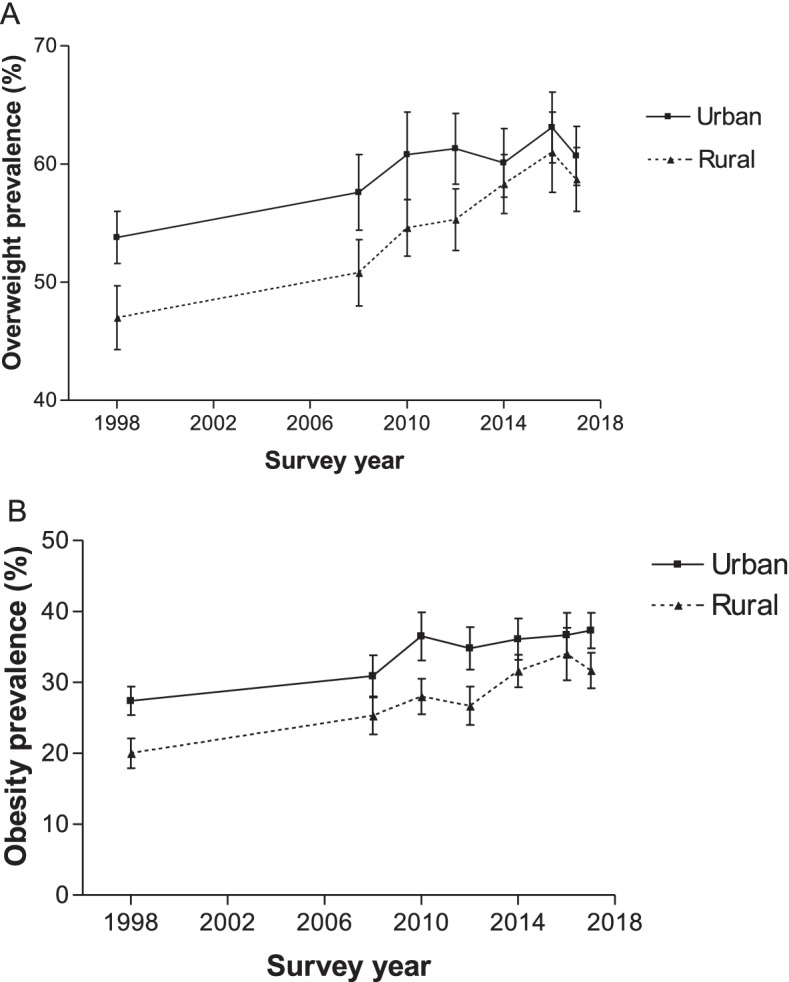
Overweight and obesity prevalence according to urban and rural residence for women aged 15–49 years from 1998 to 2017, South Africa. Error bars represent 95% confidence intervals

The urban-rural disparity in overweight and obesity prevalence decreased steadily between 1998 and 2017. As shown in Fig. 5, the overweight prevalence among urban residents steadily increased between 1998 and 2016 (from 53.8 to 63.1%; *p* < 0.0001) but fell slightly in 2017 (60.7%) to a level lower than in 2016 (*p* = 0.0095). However, the differences in prevalence estimates between 1998 and 2017 were statistically significant (*p* < 0.0001). While overweight prevalence among rural residents also steadily increased between 1998 and 2016 (from 47.0 to 61.0%; p < 0.0001), it had fallen slightly in 2017 (58.7%) to a level lower than in 2016 (*p* = 0.0370), with the difference of prevalence estimates between the 1998 and 2017 were statistically significant (*p* < 0.0001). The obesity prevalence among urban residents steadily increased between 1998 and 2016 (from 27.4 to 36.7%; p < 0.0001), but dropped in 2017 (37.3%) to a level lower than in 2017 (*p* = 0.5116). Again, the differences in prevalence estimates between 1998 and 2017 were statistically significant (*p* < 0.0001) (Fig. 5). The obesity prevalence among rural residents also steadily increased between 1998 and 2016 (from 20.0 to 34.0%; p < 0.0001), but fell slightly in 2017 (31.7%) to a level lower than in 2016 (*p* = 0.0363). Again, the differences in prevalence estimates between 1998 and 2017 were statistically significant (p < 0.0001).

### Prevalence stratified by smoking status

**Fig. 6:**
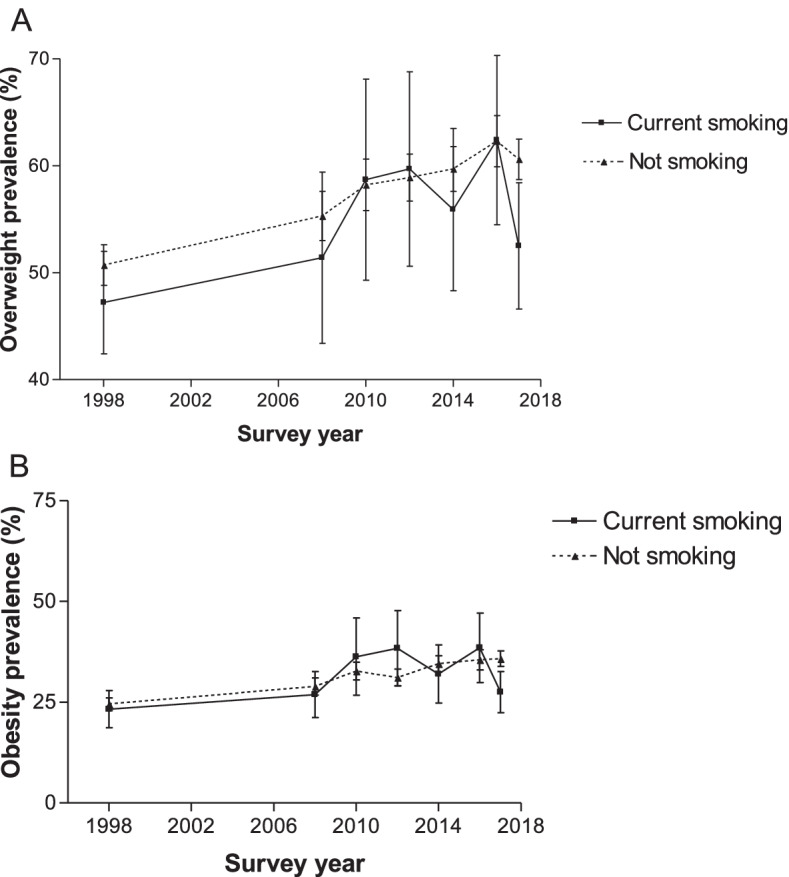
Overweight and obesity prevalence according to current smoking status for women aged 15–49 years from 1998 to 2017, South Africa. Error bars represent 95% confidence intervals

Generally, except for a few years, the overweight and obesity prevalence among WCBA currently smoking and those not smoking increased significantly between 1998 and 2017 (Fig. 6). For example, between 1998 and 2017, the obesity prevalence among WCBA currently smoking rose from 23.3 to 27.5% (*p* < 0.0001) and from 24.5 to 35.8% (p < 0.0001) among those not smoking.

Over the period, overweight and obesity prevalence was generally lower among those currently smoking than those not smoking. In some cases, overweight and obesity prevalence was similar among WCBA currently smoking and not smoking (Fig. 6).

### Prevalence stratified by socioeconomic status

**Fig. 7:**
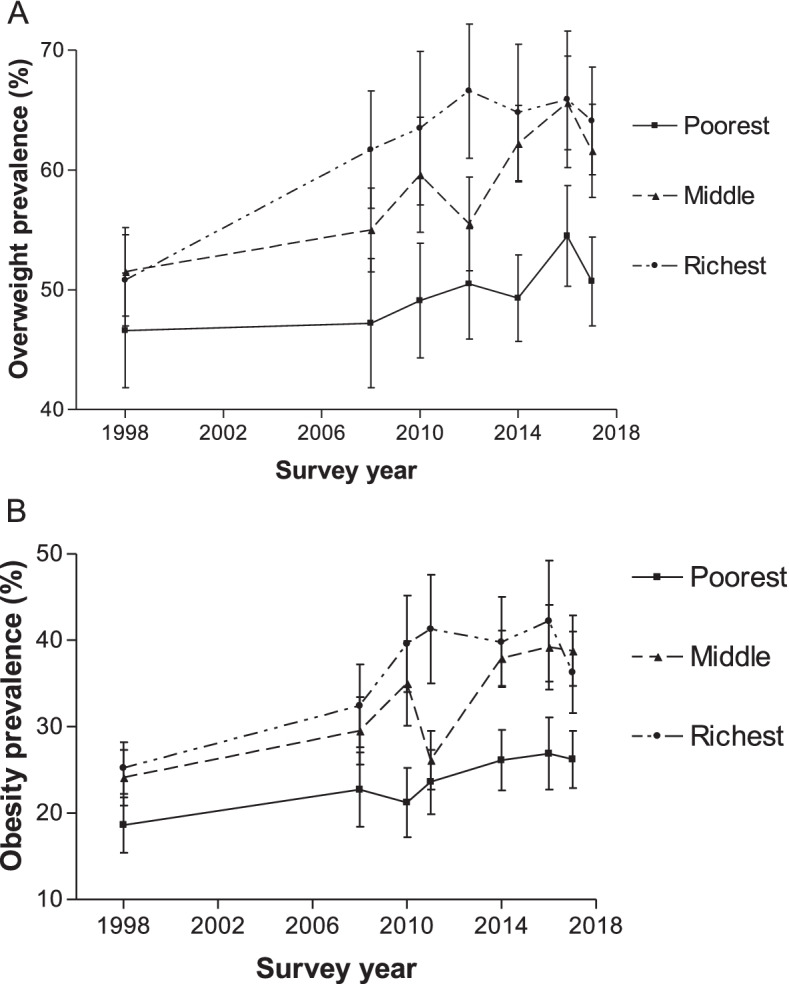
Overweight and obesity prevalence according to socioeconomic status quintile for women aged 15–49 years from 1998 to 2017, South Africa. Error bars represent 95% confidence intervals

The differences in the prevalence of overweight and obesity were more pronounced between the lowest and highest socioeconomic quintiles than between the middle and highest socioeconomic quintiles. As shown in Fig. 7, the overweight prevalence among women in the lowest socioeconomic quintile increased from 46.6% in 1998 to 54.5% in 2016 (*p* < 0.0001). The prevalence dropped in 2017 (50.7%) to a level lower than in 2016 (*p* = 0.0004), although there were no statistically significant differences for some periods. Also, overweight prevalence among women in the highest socioeconomic quintile increased from 50.8% in 1998 to 65.9% in 2016 (p < 0.0001) but decreased slightly in 2017 (64.1%) to a level lower than it was in 2016 (*p* = 0.03). A similar pattern existed for obesity prevalence across the socioeconomic quintiles. For example, among women in the lowest socioeconomic quintile, obesity prevalence appeared to be on an upward trend between 1998 and 2016 (18.6% in 1998 to 26.9% in 2016; p < 0.0001). The obesity prevalence in 2017 (26.9%) had since decreased to a level lower than it was in 2016, but not statistically significant (*p* = 0.4455). Across all the socioeconomic quintiles, the obesity prevalence in 2017 was significantly higher than the estimates in 1998 (p < 0.0001).

### Determinants of overweight and obesity

#### Age group

The odds of being overweight and obese were significantly higher with increasing age (Tables 3 and 4). For example, in 1998, women in all the older age groups (25–34 years and 35–49 years age groups), compared to those in the 15–24 years age group, had higher odds of being overweight (25–34 years versus 15–24 years, AOR 3.11, 95% CI 2.60–3.72; 35–49 years versus 15–24 years, AOR 6.60, 95% CI 5.39–8.09) (Table 3). Similarly, the odds of being obese are higher in all the older age groups compared to the 15–49 years age group (25–34 years versus 15–24 years, AOR .71, 95% CI 2.91–4.71; 35–49 years versus 15–24 years, AOR 6.87, 95% CI 5.40–8.73) (Table 4).

**Table 3:** Determinants of overweight among women of childbearing age in South Africa

	Survey year AOR (95% CI)						
Determinants	1998	2008	2010/2011	2012	2014/2015	2016	2017
**Age**							
15–24	1	1	1	1	1	1	1
25–34	3.11***	2.54***	2.83***	3.19***	3.08***	2.99***	3.51***
	(2.60–3.72)	(2.02–3.18)	(2.27–3.54)	(2.67–3.82)	(2.59–3.65)	(2.39–3.73)	(3.00–4.11)
35–49	6.60***	5.35***	5.24***	6.64***	5.65***	5.98***	5.28***
	(5.39–8.09)	(4.32–6.63)	(4.14–6.63)	(5.44–8.11)	(4.72–6.75)	(4.54–7.87)	(4.52–6.17)
**Population group**							
Non-Black African	1	1	1	1	1	1	1
Black African	1.69***	1.04	1.39	1.34	1.25	1.42*	1.12
	(1.35–2.11)	(0.73–1.48)	(0.95–2.04)	(0.98–1.83)	(0.94–1.67)	(1.01–2.00)	(0.89–1.41)
**Education**							
No schooling/Primary	1	1	1	1	1	1	1
Secondary	0.97	1.30*	1.43**	1.78***	1.36**	1.25	1.30*
	(0.82–1.15)	(1.05–1.62)	(1.14–1.79)	(1.36–2.32)	(1.11–1.66)	(0.91–1.72)	(1.03–1.64)
Tertiary	0.56***	1.33	1.39	1.71**	1.70***	1.10	1.28
	(0.41–0.76)	(0.93–1.88)	(0.99–1.96)	(1.21–2.42)	(1.24–2.33)	(0.70–1.73)	(0.95–1.73)
**Area of residence**							
Rural	1	1	1	1	1	1	1
urban	1.25*	1.15	1.16	1.10	0.92	0.92	0.98
	(1.02–1.53)	(0.90–1.45)	(0.92–1.47)	(0.91–1.34)	(0.76–1.11)	(0.73–1.16)	(0.81–1.19)
Current smoking status	0.61***	0.63**	0.81	0.86	0.73	0.92	0.62**
	(0.48–0.79)	(0.45–0.88)	(0.48–1.39)	(0.49–1.52)	(0.50–1.07)	(0.60–1.42)	(0.47–0.84)
**Socioeconomic quintiles**							
1 (poorest)	1	1	1	1	1	1	1
2 (poor)	1.12	1.07	1.47**	1.24	1.16	1.32	1.19
	(0.84–1.49)	(0.82–1.39)	(1.13–1.91)	(0.99–1.55)	(0.93–1.44)	(0.99–1.77)	(0.98–1.43)
3 (middle)	1.15	1.16	1.35	0.96	1.54***	1.59**	1.30*
	(0.87–1.53)	(0.87–1.54)	(0.99–1.83)	(0.74–1.24)	(1.24–1.91)	(1.18–2.13)	(1.03–1.63)
4 (rich)	1.27	1.36	1.26	1.32	1.65***	1.56*	1.66***
	(0.92–1.75)	(0.99–1.87)	(0.91–1.75)	(0.99–1.76)	(1.26–2.18)	(1.09–2.22)	(1.30–2.12)
5 (richest)	1.35	1.24	1.38	1.40	1.39*	1.62*	1.39*
	(0.95–1.94)	(0.79–1.96)	(0.92–2.07)	(0.98–2.02)	(1.00–1.92)	(1.06–2.46)	(1.04–1.87)
Constant	0.21***	0.35***	0.25***	0.21***	0.29***	0.30***	0.34***
	(0.15–0.30)	(0.22–0.54)	(0.15–0.41)	(0.14–0.33)	(0.20–0.43)	(0.19–0.50)	(0.24–0.48)
Observations	4993	5785	6286	7594	9034	3262	9342

*AOR* Adjusted odds ratio, 95% CI 95% confidence intervals in parentheses

*** *p* < 0.001, ** *p* < 0.01, * *p* < 0.05

**Table 4:** Determinants of obesity among women of childbearing age in South Africa

`	Survey year AOR (95% CI)						
Determinants	1998	2008	2010/2011	2012	2014/2015	2016	2017
**Age**							
15–24	1	1	1	1	1	1	1
25–34	3.71***	2.79***	3.24***	3.74***	3.65***	3.24***	3.95***
	(2.91–4.71)	(2.17–3.59)	(2.55–4.12)	(3.01–4.65)	(3.01–4.42)	(2.42–4.33)	(3.28–4.75)
35–49	6.87***	6.46***	5.88***	6.53***	6.34***	6.61***	5.86***
	(5.40–8.73)	(5.05–8.28)	(4.70–7.37)	(5.28–8.06)	(5.17–7.77)	(5.10–8.57)	(4.89–7.02)
**Population group**							
Non-Black African	1	1	1	1	1	1	1
Black African	1.71***	1.21	1.31	1.46*	0.98	1.52*	1.03
	(1.33–2.19)	(0.86–1.70)	(0.91–1.91)	(1.01–2.11)	(0.73–1.31)	(1.01–2.29)	(0.82–1.30)
**Education**							
No schooling/Primary	1	1	1	1	1	1	1
Secondary	0.90	1.11	1.29*	1.34*	1.06	1.19	1.03
	(0.73–1.10)	(0.91–1.35)	(1.04–1.60)	(1.07–1.67)	(0.86–1.30)	(0.87–1.63)	(0.82–1.30)
Tertiary	0.59**	0.93	1.29	1.38*	1.24	1.00	1.07
	(0.40–0.85)	(0.67–1.27)	(0.94–1.78)	(1.01–1.88)	(0.92–1.66)	(0.65–1.55)	(0.80–1.41)
**Area of residence**							
Rural	1	1	1	1	1	1	1
urban	1.32*	1.23	1.29*	1.24	1.09	0.88	1.23*
	(1.03–1.70)	(0.95–1.59)	(1.03–1.63)	(0.99–1.54)	(0.88–1.35)	(0.69–1.13)	(1.00–1.52)
Current smoking status	0.68*	0.73	0.87	1.21	0.65*	1.12	0.57***
	(0.50–0.93)	(0.50–1.07)	(0.51–1.50)	(0.74–1.97)	(0.44–0.96)	(0.70–1.78)	(0.43–0.74)
**Socioeconomic quintiles**							
1 (poorest)	1	1	1	1	1	1	1
2 (poor)	1.24	1.13	1.85***	1.23	1.09	1.40	1.28*
	(0.89–1.72)	(0.82–1.56)	(1.36–2.52)	(0.96–1.56)	(0.86–1.38)	(1.00–1.96)	(1.03–1.58)
3 (middle)	1.47*	1.23	1.77**	0.89	1.49**	1.84***	1.45**
	(1.04–2.07)	(0.90–1.68)	(1.26–2.48)	(0.67–1.18)	(1.16–1.93)	(1.29–2.63)	(1.10–1.91)
4 (rich)	1.76**	1.37	1.70**	1.47*	1.34	1.79**	1.60***
	(1.22–2.53)	(0.94–1.99)	(1.16–2.50)	(1.07–2.01)	(0.96–1.87)	(1.22–2.61)	(1.24–2.06)
5 (richest)	1.74**	1.27	1.78**	1.63*	1.26	2.19**	1.15
	(1.18–2.56)	(0.80–2.04)	(1.17–2.70)	(1.09–2.45)	(0.87–1.82)	(1.33–3.59)	(0.84–1.57)
Constant	0.042***	0.085***	0.060***	0.057***	0.12***	0.073***	0.11***
	(0.027–0.063)	(0.053–0.14)	(0.037–0.097)	(0.036–0.089)	(0.084–0.19)	(0.041–0.13)	(0.076–0.16)
Observations	4993	5785	6286	7594	9034	3262	9342

*AOR* Adjusted odds ratio, 95% CI 95% confidence intervals in parentheses

*** *p* < 0.001, ** *p* < 0.01, * *p* < 0.05

### Population group

In 1998 and 2016, women who self-identified as Black African had higher odds of being overweight and obese than those who self-identified as non-Black African (Tables 3 and 4).

### Education

In most years (except for 1998 and 2016), the odds of being overweight and/or obese were higher in women having a secondary education than in the reference category (no schooling/primary school education) (Tables 3 and 4). In 1998, the odds of being overweight and obese was lower in women having a tertiary education compared to those having no schooling/primary school education. Whereas in 2012 and 2014/2015, women having tertiary education, compared to no schooling/primary school education, had a higher odds of being overweight and/or obese.

### Socioeconomic status

The odds of being overweight and obese increased with wealth as women living in wealthier households had greater odds of being overweight or obese than those living in households with lower socioeconomic status (Tables 3 and 4).

### Current smoking status

In general, smoking was inversely associated with overweight and obesity. Smoking reduced the odds of being overweight and/or obese (Tables 3 and 4). Compared to women residing in rural areas, those residing in urban areas had significantly greater odds of being overweight (AOR 1.25, 95% CI 1.02–1.53) and obese (AOR 1.32, 95% CI 1.03–1.70) in 1998. In 2017, women residing in rural areas compared with those residing in urban areas had significantly greater odds of being obese (AOR 1.23, 95% CI 1.00–1.52). The effect of urbanicity on overweight and obesity was not statistically significant for the other years.

## Discussion

This study assessed the changes in the prevalence of overweight and obesity between 1998 to 2017 for non-pregnant women aged 15 to 49 years in South Africa. It also examined the determinants of overweight and obesity. The paper found a general upward trend in overweight prevalence from 51.3 to 60.0% and obesity from 24.7 to 35.2% over the period. Overweight and obesity prevalence remained higher for older than younger women. In 1998, women with no schooling/primary education and those with secondary education had a higher overweight and obesity prevalence than those with tertiary education. This pattern was reversed in 2017. Also, the prevalence of overweight and obesity tended to be higher among women from wealthier socioeconomic backgrounds than their counterparts from less wealthy backgrounds. For most women, the prevalence of overweight and/or obesity in 2017 was significantly higher than the estimate in 1998. Significant predictors of overweight and obesity included increased age, self-identifying with the Black African population group, higher educational attainment, residing in an urban area, and wealth. Smoking was inversely associated with being overweight and obese.

South Africa is undergoing a nutrition transition, characterised by an increasing prevalence of overweight and obesity. Our finding that overweight and obesity increased over time was consistent with previous studies from sub-Saharan Africa [27, 31–34]. Many factors could account for this rise in prevalence over time in South Africa, including rapid economic development since the new democracy in 1994, urbanisation and increased female labour force participation (i.e. working outside the home) [57]. Working women tend to have low-energy jobs, and mobility is less energy-intensive because of shorter commutes and the use of motorised transportation. Furthermore, time constraint is a challenge for many women in preparing healthy meals because of long working hours and having greater access to processed foods. The South African National Health and Nutrition Examination Survey (SANHANES) [41] indicated that more older men and women ate outside their homes every month than their younger counterparts (24.1% for 15–24 years; 25.9% for 15–34 years, 32.2% for 35–44 years and 33.4% for 45–54 years). The Growth, Employment and Redistribution (GEAR) Policy in 1996 liberalised the South African economy, leading to the rapidly changing food environment. This significantly increased the number of large transnational food and beverage industries, supermarkets and fast-food chains [58–60]. These contributed to the widespread availability and acceptability of cheap processed foods; people changed diets from traditional to Western lifestyle diets with more processed high energy-dense foods of poor quality and low nutritional value coupled with increased sedentary lifestyles [8, 9].

Consistent with previous studies from sub-Saharan Africa, overweight and obesity prevalence varies by age groups, educational attainment, urban/rural residence, socioeconomic status, race/ethnicity [31–33]. Similar to previous studies from South Africa and elsewhere [25, 28], this paper found that the odds of being overweight and obese were significantly higher with increasing age. This relationship was consistent over time and could be due, in part, to the increased physical inactivity among older women and increased weight gain during this life stage [36, 61, 62]. Also, increased consumption of unhealthy food and convenient foods (i.e. food prepared outside the home, takeaways, and readymade meals) as discussed above may compound overweight and obesity in women in South Africa during this life stage [63, 64].

This study finds that in 1998 and 2016, the odds of being overweight and obese was greater among women who self-identified as Black African population group than the non-Black African population group. This observed relationship may be partly due to not only nutrition transition [8, 9] but to other factors, including the impact of changes in the food environment^1^ towards unhealthy eating [66] for the different population groups residing in neighbourhoods perceived to be unsafe (which limits ability and willingness to engage in physical activities), culture, socioeconomic status and the built environment (which constitute obstacles to physical activity) [67]. In addition, the observation might be due to the perception of larger body size as a sign of wealth in the Black African population group [67].

Our study finds that, in 2012 and 2014/2015, women having tertiary education, compared to no schooling/primary school education, had a higher odds of being overweight and/or obese. In addition, in most years (except for 1998 and 2016), the odds of being overweight and/or obese was higher in women having a secondary education compared to those with no schooling/primary school education. This corroborates other studies from sub-Saharan Africa [68–72]. Those with higher education tend to have less energy-demanding jobs, be more physically inactive and have sedentary lifestyles [73]. By contrast, we find that in 1998, the odds of being overweight and obese was lower in women having a tertiary education compared to those having no schooling/primary school education. This is consistent with the findings of Puoane and colleagues [74] that women with tertiary education had a lower BMI than those with some schooling, maybe because they are more aware of the health benefits of physical activity. Micklesfield and colleagues [67] suggested that this finding may be due to the wide distributions of education and socioeconomic status among the South African population just after the country became a democracy in 1994.

Consistent with previous studies from sub-Saharan Africa [32, 33], this paper finds that in 1998 and 2017, women residing in urban areas had a higher odds of being overweight or obese than those who resided in rural areas. The finding of a higher odds of overweight and obesity in urban areas is due to the westernised diets, processed food consumption and lifestyles, including increased physical inactivity and sedentary behaviour characteristic of the urban populations [7]. Furthermore, the SANHANES indicated that more men and women living in formal urban settlements have ever eaten outside the home (57.3%) than those living in formal rural settlements (36.4%) [41].

Also, in line with the studies from South Africa [75–77] and other sub-Saharan African countries [31–33, 36, 70, 72, 78, 79], women living in wealthier households had higher odds of being overweight and obese compared to those in lower socioeconomic groups. The relationship between socioeconomic status and overweight or obesity was consistent over time, suggesting an inverse socioeconomic gradient in overweight and obesity occurring in the context of the nutrition transition [8, 9]. While there may be the perception of larger body size as a sign of wealth [67], there is still no clear explanation for this gradient.

In general, the study found that WCBA who currently smoke had a lower prevalence of overweight and obesity than those who did not smoke. A few years were an exception to this trend; however, WCBA who currently smoke, had similar overweight and obesity prevalence to those who did not smoke. In keeping with previous studies from South Africa [40], smoking was inversely associated with being overweight and obese, which may explain its ability to increase energy expenditure and suppress appetite leading to weight loss [40, 80]. As such, smokers will need support to find alternative ways to lose weight such as exercise when quitting smoking.

The COVID-19 pandemic has highlighted the importance of caring for those with conditions such as obesity and overweight as these conditions put them at a greater risk of death and severe COVID-19 [4, 5]. With the COVID-19 pandemic and high prevalence of obesity among WCBA in South Africa, to minimise adverse consequences, there is an urgent call to prioritise the vulnerable populations through timeous vaccination, testing and detection, and providing prompt and aggressive treatment for obese patients [5] even before their conditions become severe.

### Policy implications

This study has some policy implications. The current tax on sugar-sweetened beverages [81] highlights an example of the government’s commitment to fighting non-communicable diseases, including obesity and overweight in South Africa. However, the high prevalence and pattern of overweight and obesity among WCBA reported in this paper means the government needs to complement the sugar-sweetened beverage tax with other policies to address overweight and obesity. Although many essential food items, including “healthy” food items, are exempted from value-added tax in South Africa [82], they could be further subsidised to increase accessibility and availability [2]. There is also a need for awareness-raising campaigns promoting healthy eating and lifestyles among WCBA [21], bearing in mind that the risk of obesity increases with age. Regular anthropometric measurement is crucial for confirming overweight or obesity status and for timely interventions [83]. Based on the study findings, we advocate for routine weight monitoring in WCBA to identify sub-population groups that need timely action. These women should regularly check their weight at home or during health visits [21]. Moderate to vigorous physical exercise is recommended for at least 150 min a week to maintain good health [21]. To maintain a healthy weight, women need to exercise regularly. However, having a safe physical environment for exercise is an issue in South Africa [84]. In addition to advocating for increased physical activities among WCBA, the government should secure the physical environment to enhance physical exercise, given the relatively high burden of overweight and obesity reported in this paper. The finding of a more rapidly increasing prevalence of overweight and obesity between 1998 and 2017 in rural areas compared to urban areas was consistent with previous literature [85], citing shifts from eating healthy to unhealthy food among rural residents, to be responsible for the trend. The finding suggests the need for the government to promote access (including availability and affordability) to good quality food in rural and urban areas. There is also a need for action on the social determinants of health reported in this paper to reduce the burden of obesity and overweight among WCBA in South Africa.

### Strengths and limitations of the study

This study has some strengths and limitations. The use of comparable nationally representative secondary data, covering two decades, allowed for applying sampling weights and generalising the results. Also, an objective measure of BMI was used to categorise women as overweight and obese. This study uses only non-pregnant women because BMI measures can be conflated by pregnancy. Employment as an important explanatory variable was omitted because it was not collected in the same way between these datasets. Moreover, employment is another measure of socioeconomic status that could also be correlated with, for example, education already included in the model. Some risk factors associated with overweight and obesity were not contained in the datasets. Examples of risk factors not contained in the datasets but associated with higher odds of overweight and obesity include Increased parity [38], increased television viewing or a sedentary lifestyle [38, 39].

### Recommendations for future research

We recommend future research in several areas. Further research needs to assess changes over time in socioeconomic inequality in overweight and obesity among WCBA and to decompose this inequality into determinants to identify their contribution to inequality. Future research could also be conducted to investigate whether changes in processed food consumption patterns, a likely determinant of overweight and obesity, occurred in households and explore how that affects socioeconomic inequality in overweight and obesity.

## Conclusion

In South Africa, overweight and obesity prevalence increased among non-pregnant WCBA, especially older women. Prevention of overweight and obesity by focusing on the most affected groups identified in this paper is critical to reduce the health problems. Several opportunities exist to prevent and reduce the burden of obesity in WCBA in South Africa as highlighted in this paper, including action on many determinants of health. In the context of COVID-19 and for South Africa to meet key national targets. There is a need to focus on older women, those self-identified as Black African, with higher educational attainment, residing in urban areas, and wealthy.

## Data Availability

The datasets analysed during the current study are publicly available in the DataFirst open research data repository based at the University of Cape Town [www.datafirst.uct.ac.za] and the DHS Program repository [https://www.dhsprogram.com/Data/]. Data are accessed free of charge upon registration on the DataFirst portal [86–91] and the DHS Program repository [11].

## Abbreviations

%: Percent
95% CI: 95% confidence interval
AOR: Adjusted odds ratio
BMI: Body Mass Index
COVID-19: Corona-virus disease 2019
CSM: Continuing Sample Member
EAs: Enumeration Areas
GEAR Policy: Growth, Employment and Redistribution Policy
kg: kilogram
LMICs: Low- and middle- income countries
m^2^: metre squared
NIDS: National Income Dynamics Study
SANHANES: South African National Health and Nutrition Examination Survey
SA: South Africa
SD: Standard deviation
WCBA: Women of childbearing age
WHO: World Health Organization

## Footnotes

1 The food environment refers to “the collective physical, economic, policy and socio-cultural surroundings, opportunities and conditions that influence people’s food and beverage choices and nutritional status ”[65]
